# Mid‐Term Clinical Outcomes According to CTO Revascularization Status in Patients With NSTEMI and Multivessel Disease

**DOI:** 10.1002/clc.70491

**Published:** 2026-10-22

**Authors:** Wei Liu, Yuling Li, Wei Qin, Jixian Gao, Bo Liu, Chongyou Rao, Bing Han

**Affiliations:** ^1^ Department of Cardiology, Xuzhou Central Hospital Xuzhou Clinical College of Xuzhou Medical University Xuzhou Jiangsu China; ^2^ Department of Cardiology, Xuzhou Central Hospital Southeast University Xuzhou Jiangsu China; ^3^ Department of Ultrasonography, Xuzhou Central Hospital Southeast University Xuzhou Jiangsu China; ^4^ Department of Cardiology Affiliated Hospital of Chengde Medical University Chengde Hebei China; ^5^ Department of Cardiology Cangzhou People's Hospital Cangzhou Hebei China; ^6^ Department of Information Center Yuncheng Central Hospital Yuncheng Shanxi China; ^7^ Medical Innovation Research Division Chinese PLA General Hospital Beijing China

**Keywords:** chronic total occlusion, clinical outcomes, multivessel coronary artery disease, non–ST‐segment elevation myocardial infarction, percutaneous coronary intervention, revascularization

## Abstract

**Background:**

The clinical relevance of chronic total occlusion (CTO) revascularization in patients with non–ST‐segment elevation myocardial infarction (NSTEMI) and multivessel disease (MVD) remains uncertain.

**Methods:**

In this multicenter retrospective cohort study, 632 patients with NSTEMI, MVD, and CTO were included. Patients were categorized into a complete revascularization group involving successful CTO recanalization (CR‐CTO, *n* = 119) and an incomplete revascularization group without successful CTO recanalization (ICR‐CTO, *n* = 513). The primary endpoints were major adverse cardiovascular and cerebrovascular events (MACCE) and major adverse cardiovascular events (MACE).

**Results:**

During a mean follow‐up of 528 ± 201 days, the incidences of MACCE (11.8% vs. 28.5%) and MACE (7.6% vs. 24.2%) were significantly lower in the CR‐CTO group than in the ICR‐CTO group (both *p* < 0.001). In multivariable Cox regression analyses, CR‐CTO was independently associated with lower risks of MACCE (hazard ratio [HR], 0.35; 95% confidence interval [CI], 0.20–0.61) and MACE (HR, 0.26; 95% CI, 0.13–0.51). Among patients without successful CTO recanalization, additional revascularization of non‐CTO lesions was not associated with improved outcomes.

**Conclusions:**

In patients with NSTEMI, MVD, and CTO, complete revascularization involving successful CTO recanalization was associated with improved mid‐term clinical outcomes. In contrast, additional revascularization of non‐CTO lesions alone was not associated with a prognostic benefit when CTO lesions remained unrevascularized. These findings suggest that CTO revascularization status may warrant specific consideration when revascularization strategies are evaluated in this patient population.

**Abbreviations**
AMI: acute myocardial infarction
CABG: coronary artery bypass grafting
CAG: coronary angiography
CI: confidence interval
CR‐CTO: complete revascularization involving chronic total occlusion lesions
CR‐nCTO: complete revascularization of non‐chronic total occlusion lesions
CTO: chronic total occlusion
HDL‐C: high‐density lipoprotein cholesterol
HR: hazard ratio
ICR‐CTO: incomplete revascularization involving chronic total occlusion lesions
ICR‐nCTO: incomplete revascularization of non‐chronic total occlusion lesions
IQR: interquartile range
LDL‐C: low‐density lipoprotein cholesterol
LMCA: left main coronary artery
MACCE: major adverse cardiovascular and cerebrovascular events
MACE: major adverse cardiovascular events
MVD: multivessel disease
NSTEMI: non‐ST‐segment elevation myocardial infarction
OMT: optimal medical therapy
PCI: percutaneous coronary intervention
STEMI: ST‐segment elevation myocardial infarction
TIMI: thrombolysis in myocardial infarction

## 1 Introduction

Patients with non‐ST‐segment elevation myocardial infarction (NSTEMI) and multivessel disease (MVD) remain at high risk of recurrent ischemic events and adverse long‐term outcomes despite advances in contemporary revascularization strategies [1, 2]. Chronic total occlusion (CTO) is one of the most complex lesion subsets in patients with MVD. Because of complex coronary anatomy and technical challenges, CTO lesions remain a major determinant of incomplete revascularization in patients undergoing percutaneous coronary intervention (PCI) [3].

In recent years, advances in interventional devices, refinement of procedural techniques, and increasing operator experience have substantially improved the success rates of CTO percutaneous coronary intervention (CTO‐PCI) [4, 5, 6]. Consequently, CTO‐PCI has become an increasingly established revascularization option in selected patients. However, whether CTO‐PCI is associated with improved clinical outcomes remains controversial in real‐world practice [7, 8, 9, 10, 11, 12, 13].

Crucially, most previous studies evaluating the prognostic impact of CTO in patients with acute myocardial infarction (AMI) and MVD have primarily focused on STEMI populations, whereas data specific to NSTEMI remain

sparse and are often confined to retrospective subgroup analyses [14, 15, 16, 17, 18]. In particular, whether additional non‐CTO lesion revascularization improves prognosis when CTO lesions remain unrevascularized remains uncertain. To date, large‐scale multicenter studies specifically addressing this issue in NSTEMI patients remain limited.

Therefore, this multicenter retrospective cohort study aimed to evaluate the association between CTO revascularization status and mid‐term clinical outcomes in patients with NSTEMI and MVD. We also explored whether additional revascularization of non‐CTO lesions was associated with improved outcomes among patients in whom CTO recanalization was not achieved.

## 2 Methods

### 2.1 Study Design and Population

This multicenter retrospective cohort study consecutively enrolled patients with NSTEMI from five tertiary medical centers in China between January 1, 2021, and December 31, 2022. A total of 4490 patients with NSTEMI were initially screened.

The inclusion criteria were as follows: (1) age ≥ 18 years; (2) confirmed NSTEMI with coronary angiography performed during the index hospitalization; and (3) MVD with at least one CTO involving a major epicardial coronary artery. The exclusion criteria were as follows: (1) in‐hospital death; (2) prior coronary artery bypass grafting (CABG); and (3) malignancy with an expected survival of < 1 year. Because the present study focused on post‐discharge mid‐term outcomes, patients who died during the index hospitalization were excluded. Guideline‐directed medical therapy was recommended according to contemporary clinical practice and guideline recommendations.

This study was conducted in accordance with the principles of the Declaration of Helsinki and was approved by the Biomedical Research Ethics Review Committee of Xuzhou Central Hospital (approval No. XZXY‐LJ‐20200415‐074). Given the retrospective observational design of the study and the use of anonymized clinical data, the requirement for written informed consent was waived by the ethics committee. The study was reported in accordance with the Strengthening the Reporting of Observational Studies in Epidemiology (STROBE).

### 2.2 Definitions and Study Endpoints

NSTEMI was diagnosed based on clinical symptoms, electrocardiographic changes, elevated cardiac troponin levels, and CAG findings [19]. MVD was defined as ≥ 70% stenosis in at least two major epicardial coronary arteries or ≥ 50% stenosis in the left main coronary artery (LMCA) [20]. CTO was defined as TIMI flow grade 0 with an estimated duration ≥ 3 months and without evidence of acute thrombosis, plaque rupture, or erosion [7]. Major epicardial coronary arteries included the left anterior descending artery, left circumflex artery, and right coronary artery.

Revascularization decisions were made by the treating interventional cardiologists or heart team according to lesion characteristics, clinical status, patient preference, and current guidelines [21, 22]. Complete revascularization involving CTO lesions (CR‐CTO) was defined as successful recanalization of CTO lesions with complete treatment of clinically significant non‐CTO lesions, when applicable. Incomplete revascularization involving CTO lesions (ICR‐CTO) was defined as the absence of successful CTO recanalization, regardless of whether non‐CTO lesions were treated. Successful CTO revascularization was defined as residual stenosis < 30% with final TIMI flow grade ≥ 2 after PCI or complete target‐vessel revascularization by CABG.

The primary endpoints were major adverse cardiovascular and cerebrovascular events (MACCE) and major adverse cardiovascular events (MACE). MACCE included all‐cause death, recurrent myocardial infarction, heart failure, stroke, and urgent revascularization. MACE included cardiovascular death, recurrent myocardial infarction, heart failure, and urgent revascularization.

### 2.3 Follow‐Up and Outcome Assessment

Follow‐up data were obtained through outpatient visits, telephone interviews, and hospitalization records. Patients were considered lost to follow‐up if contact could not be established after three attempts and no subsequent hospitalization records were available.

Clinical events were adjudicated based on available medical records and follow‐up information. Recurrent myocardial infarction was defined according to guideline‐based diagnostic criteria. Heart failure events were defined as heart failure requiring hospitalization or intensification of diuretic therapy. Stroke included ischemic or hemorrhagic stroke confirmed by clinical assessment and imaging findings. Urgent revascularization was defined as unplanned revascularization requiring urgent PCI or CABG because of recurrent ischemia or acute coronary events.

### 2.4 Statistical Analysis

Continuous variables are presented as mean ± standard deviation (SD), unless otherwise specified. Categorical variables are presented as counts and percentages. Between‐group comparisons were performed using Student's *t*‐test for continuous variables and the chi‐square test or Fisher's exact test for categorical variables, as appropriate. Kaplan–Meier curves and log‐rank tests were used to compare cumulative event‐free survival between groups. Cox proportional hazards models were used to evaluate the association between revascularization status and clinical outcomes. Multivariable models were adjusted for clinically relevant baseline variables. Subgroup analyses within the ICR‐CTO population were performed using the same modeling strategy. The proportional hazards assumption was assessed using Schoenfeld residuals. Variables with > 20% missing data were excluded from multivariable analyses. Statistical analyses were performed using RStudio version 4.1.3. A two‐sided *p* value < 0.05 was considered statistically significant.

## 3 Results

### 3.1 Patient Selection and Study Flow

A total of 4490 patients with NSTEMI who underwent coronary angiography between January 2021 and December 2022 were initially screened. After application of the inclusion and exclusion criteria, 2651 patients with MVD were eligible for follow‐up. Among them, 191 patients were lost to follow‐up, resulting in a final study population of 2460 patients.

Among the included patients, 632 had concomitant CTO. According to CTO revascularization status, 119 patients were classified into the complete revascularization group involving CTO lesions (CR‐CTO), and 513 were classified into the incomplete revascularization group without successful CTO recanalization (ICR‐CTO). The study flow is shown in Figure 1.

**Figure 1:**
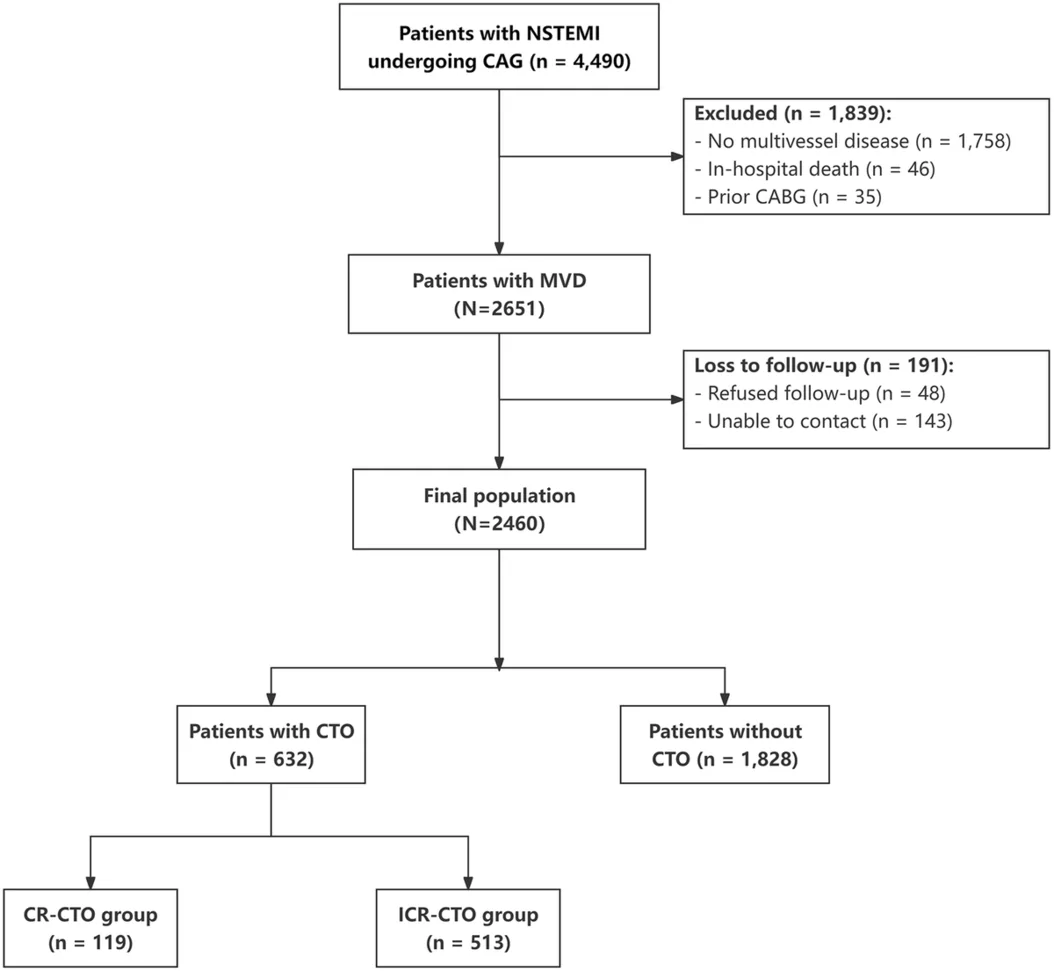
Study flow diagram of patient selection and group assignment. CAG, coronary angiography; CR‐CTO, complete revascularization including CTO lesions; CTO, chronic total occlusion; ICR‐CTO, incomplete revascularization including CTO lesions; MVD, multivessel disease; NSTEMI, non–ST‐segment elevation myocardial infarction.

### 3.2 Baseline Characteristics

Baseline characteristics are summarized in Table 1. Compared with the ICR‐CTO group, patients in the CR‐CTO group had higher high‐density lipoprotein cholesterol levels and a lower prevalence of coronary calcification (both *p* < 0.05). Other baseline characteristics were generally comparable between groups.

**Table 1:** Baseline characteristics of patients according to CTO revascularization status.

Variable	Overall (*n* = 632)	ICR‐CTO (*n* = 513)	CR‐CTO (*n* = 119)	*p* value
*Demographics*				
Age, years [median (IQR)]	66.0 (57.0–73.0)	67.0 (57.0–74.0)	65.0 (57.0–71.5)	0.089
Male sex, *n* (%)	451 (71.4)	365 (71.2)	86 (72.3)	0.896
*Comorbidities*				
Hypertension, *n* (%)	407 (64.4)	335 (65.3)	72 (60.5)	0.380
Diabetes mellitus, *n* (%)	273 (43.2)	218 (42.5)	55 (46.2)	0.525
Dyslipidemia, *n* (%)	357 (56.5)	291 (56.7)	66 (55.5)	0.883
Ischemic stroke, *n* (%)	130 (20.6)	109 (21.2)	21 (17.6)	0.454
Chronic kidney disease, *n* (%)	133 (21.0)	108 (21.1)	25 (21.0)	> 0.99
Atrial fibrillation, *n* (%)	28 (4.4)	22 (4.3)	6 (5.0)	0.910
Valvular heart disease, *n* (%)	10 (1.6)	6 (1.2)	4 (3.4)	0.187
Peripheral atherosclerotic disease, *n* (%)	25 (4.0)	17 (3.3)	8 (6.7)	0.145
*Medical history and lifestyle*				
Prior myocardial infarction, *n* (%)	107 (16.9)	93 (18.1)	14 (11.8)	0.125
Current smoker, *n* (%)	243 (38.4)	206 (40.2)	37 (31.1)	0.084
Family history of coronary artery disease, *n* (%)	44 (7.0)	34 (6.6)	10 (8.4)	0.627
*Laboratory parameters*				
eGFR, mL/min/1.73 m^2^ (median [IQR])	75.1 (57.3–93.8)	73.5 (57.2–91.8)	78.5 (59.7–96.8)	0.492
Creatine kinase, U/L (median [IQR])	143.0 (81.0–334.0)	143.0 (81.0–319.0)	140.0 (85.0–411.0)	0.546
Hemoglobin, g/L (median [IQR])	137.0 (125.0–148.0)	136.0 (124.0–147.0)	139.0 (128.0–151.0)	0.086
Platelet count, ×10^9^/L (median [IQR])	212.0 (175.0–255.0)	212.0 (175.0–255.0)	213.0 (177.0–256.0)	0.885
Albumin, g/L (median [IQR])	40.3 (37.5–43.1)	40.2 (37.6–43.1)	40.5 (37.2–43.1)	0.767
Glucose, mmol/L (median [IQR])	6.41 (5.11–8.77)	6.42 (5.07–8.80)	6.34 (5.20–8.11)	0.844
Triglycerides, mmol/L (median [IQR])	1.35 (0.99–1.97)	1.37 (1.00–1.94)	1.33 (0.99–2.23)	0.998
Total cholesterol, mmol/L (median [IQR])	4.10 (3.35–4.82)	4.04 (3.31–4.80)	4.28 (3.48–5.01)	0.130
HDL‐C, mmol/L (median [IQR])	0.99 (0.84–1.15)	0.97 (0.83–1.15)	1.06 (0.88–1.23)	0.011
LDL‐C, mmol/L (median [IQR])	2.52 (1.92–3.18)	2.50 (1.91–3.17)	2.72 (1.98–3.25)	0.286
*Cardiac function and clinical scores*				
LVEF, % (median [IQR])	54.0 (44.0–58.0)	53.0 (44.0–58.0)	55.0 (43.5–58.5)	0.637
Killip class, *n* (%)				0.892
Class I	496 (78.5)	401 (78.2)	95 (79.8)	—
Class II	97 (15.3)	81 (15.8)	16 (13.4)	—
Class III	27 (4.3)	21 (4.1)	6 (5.0)	—
Class IV	12 (1.9)	10 (1.9)	2 (1.7)	—
GRACE score (median [IQR])	144.0 (123.8–167.0)	146.0 (126.0–170.0)	141.0 (120.0–160.0)	0.111
*Angiographic characteristics*				
Two‐vessel disease, *n* (%)	212 (33.5)	179 (34.9)	33 (27.7)	0.167
Three‐vessel disease, *n* (%)	420 (66.5)	334 (65.1)	86 (72.3)	0.167
Coronary calcification, *n* (%)	113 (17.9)	102 (19.9)	11 (9.2)	0.009
CTO lesion location, *n* (%)				0.456
Left anterior descending artery	185 (29.3)	145 (28.3)	40 (33.6)	—
Right coronary artery	232 (36.7)	189 (36.8)	43 (36.1)	—
Left circumflex artery	215 (34.0)	179 (34.9)	36 (30.3)	—
Left main stenosis ≥ 50%, *n* (%)	76 (12.0)	65 (12.7)	11 (9.2)	0.379
*Interventional treatment*				
Drug‐coated balloon use, *n* (%)	46 (7.3)	38 (7.4)	8 (6.7)	0.950
Left main intervention, *n* (%)	11 (1.7)	10 (1.9)	1 (0.8)	0.657
LAD intervention, *n* (%)	139 (22.0)	117 (22.8)	22 (18.5)	0.367
LCX intervention, *n* (%)	116 (18.4)	102 (19.9)	14 (11.8)	0.054
RCA intervention, *n* (%)	126 (19.9)	97 (18.9)	29 (24.4)	0.224
*Medications at discharge*				
Aspirin, *n* (%)	610 (96.5)	493 (96.1)	117 (98.3)	0.362
Clopidogrel, *n* (%)	356 (56.3)	298 (58.1)	58 (48.7)	0.080
Ticagrelor, *n* (%)	268 (42.4)	208 (40.5)	60 (50.4)	0.063
Diuretics, *n* (%)	189 (29.9)	145 (28.3)	44 (37.0)	0.079
Nitrates, *n* (%)	475 (75.2)	378 (73.7)	97 (81.5)	0.096
ACEI/ARB, *n* (%)	277 (43.8)	232 (45.2)	45 (37.8)	0.172
β‐blockers, *n* (%)	488 (77.2)	394 (76.8)	94 (79.0)	0.695

Abbreviations: ACEI, angiotensin‐converting enzyme inhibitor; ARB, angiotensin receptor blocker; CR‐CTO, complete revascularization including CTO; eGFR, estimated glomerular filtration rate; HDL‐C, high‐density lipoprotein cholesterol; ICR‐CTO, incomplete revascularization including CTO; IQR, interquartile range; LDL‐C, low‐density lipoprotein cholesterol; LVEF, left ventricular ejection fraction.

### 3.3 Clinical Outcomes

Over a mean follow‐up of 528 ± 201 days, MACCE occurred less frequently in the CR‐CTO group than in the ICR‐CTO group (11.76% vs. 28.46%, *p* < 0.001). Similarly, the incidence of MACE was lower in the CR‐CTO group than in the ICR‐CTO group (7.56% vs. 24.17%, *p* < 0.001). Kaplan–Meier analysis demonstrated significantly higher MACCE‐free and MACE‐free survival in the CR‐CTO group than in the ICR‐CTO group (both log‐rank *p* < 0.001; Figure 2).

**Figure 2:**
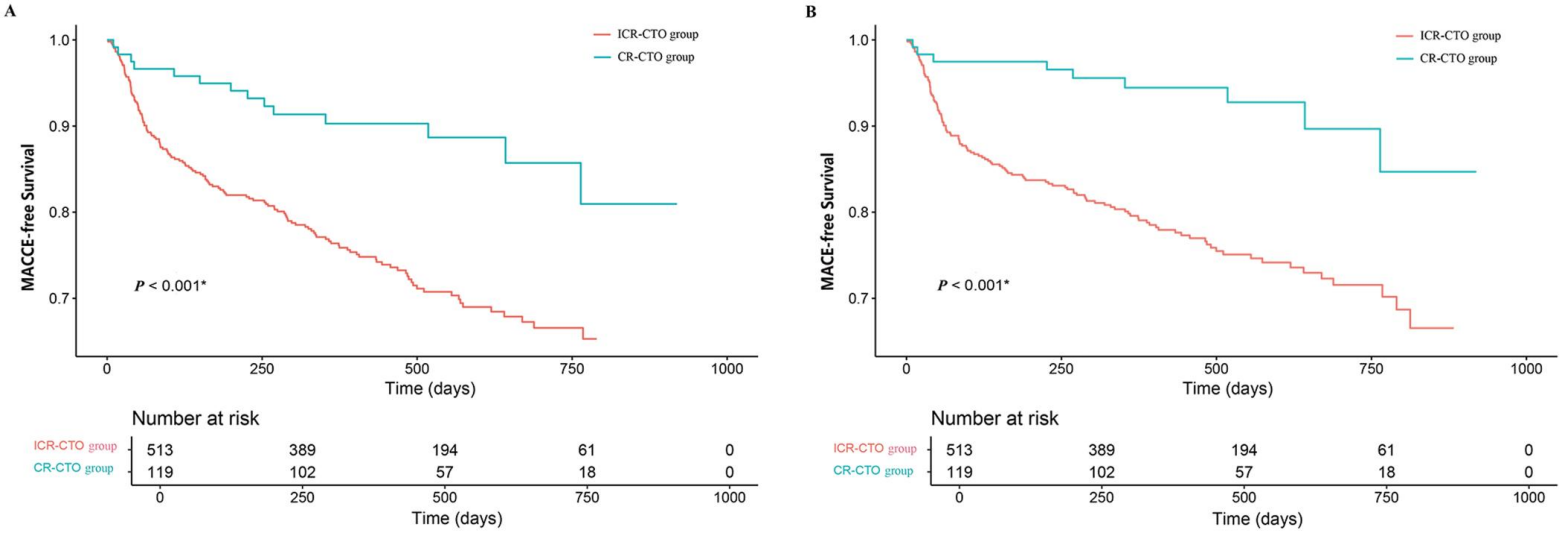
Kaplan–Meier curves for MACCE‐free survival (A) and MACE‐free survival (B) according to CTO revascularization status. CR‐CTO, complete revascularization including CTO lesions; CTO, chronic total occlusion; ICR‐CTO, incomplete revascularization including CTO lesions; MACE, major adverse cardiovascular events; MACCE, major adverse cardiovascular and cerebrovascular events.

### 3.4 Association Between CTO Revascularization Status and Clinical Outcomes

Using the ICR‐CTO group as the reference, multivariable Cox proportional hazards regression analysis showed that CR‐CTO was independently associated with lower risks of MACCE and MACE (Table 2). In the fully adjusted model, successful CTO recanalization was associated with lower risks of MACCE (hazard ratio [HR] 0.35, 95% confidence interval [CI] 0.20–0.61; *p* < 0.001) and MACE (HR 0.26, 95% CI 0.13–0.51; *p* < 0.001).

**Table 2:** Association between successful CTO recanalization and mid‐term clinical outcomes.

Endpoint	Group	Events/total	Event rate (%)	Model	HR (95% CI)	*p* value
MACCE	ICR‐CTO	146/513	28.46	Reference	1.00	—
	CR‐CTO	14/119	11.76	Model 1	0.37 (0.22–0.64)	< 0.001
				Model 2	0.38 (0.22–0.65)	< 0.001
				Model 3	0.35 (0.20–0.61)	< 0.001
MACE	ICR‐CTO	124/513	24.17	Reference	1.00	—
	CR‐CTO	9/119	7.56	Model 1	0.28 (0.14–0.56)	< 0.001
				Model 2	0.28 (0.14–0.55)	< 0.001
				Model 3	0.26 (0.13–0.51)	< 0.001

*Note:* Model 1, unadjusted; Model 2, adjusted for age and sex; Model 3, adjusted for clinically relevant baseline variables.

Abbreviations: CI, confidence interval; CTO, chronic total occlusion; HR, hazard ratio; CR‐CTO, complete revascularization including CTO; ICR‐CTO, incomplete revascularization including CTO.

### 3.5 Subgroup Analysis in the ICR‐CTO Population

Among the 513 patients in the ICR‐CTO group, none achieved successful CTO recanalization. However, the revascularization status of non‐CTO lesions varied. Specifically, 153 patients underwent complete revascularization of non‐CTO lesions (CR‐nCTO group), 155 underwent partial revascularization (ICR‐nCTO group), and 205 received optimal medical therapy alone (OMT‐nCTO group). Baseline characteristics of the non‐CTO revascularization subgroups are summarized in Supporting Information S1: Table 1.

Kaplan–Meier analysis showed no significant differences in MACCE‐free or MACE‐free survival among the OMT‐nCTO, ICR‐nCTO, and CR‐nCTO groups (both log‐rank *p* = 0.91; Figure 3). Consistent with these findings, Cox proportional hazards regression analysis demonstrated that non‐CTO lesion revascularization status was not significantly associated with the risks of MACCE or MACE (Table 3).

**Figure 3:**
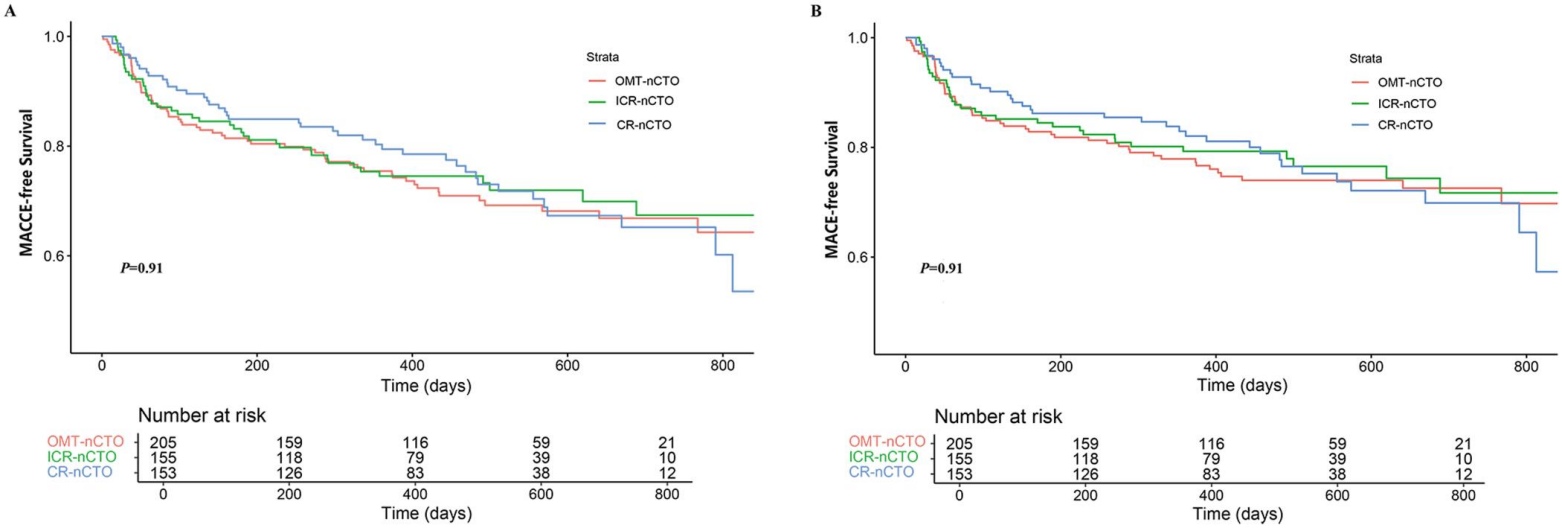
Kaplan–Meier curves for MACCE‐free survival (A) and MACE‐free survival (B) according to non‐CTO lesion revascularization status in the ICR‐CTO population. CR‐nCTO, complete revascularization of non‐CTO lesions; ICR‐nCTO, incomplete revascularization of non‐CTO lesions; MACE, major adverse cardiovascular events; MACCE, major adverse cardiovascular and cerebrovascular events; OMT‐nCTO, optimal medical therapy for non‐CTO lesions.

**Table 3:** Association between non‐CTO lesion revascularization and clinical outcomes among patients without successful CTO recanalization.

Endpoint	Group	Events/total	Event rate (%)	Model	HR (95% CI)	*p* value
MACCE	OMT‐nCTO	61/205	29.8	Reference	1.00	—
	ICR‐nCTO	42/155	27.1	Model 1	0.93 (0.63–1.38)	0.726
				Model 2	0.93 (0.63–1.38)	0.730
				Model 3	0.88 (0.59–1.33)	0.548
	CR‐nCTO	43/153	28.1	Model 1	0.92 (0.63–1.37)	0.696
				Model 2	0.93 (0.63–1.37)	0.710
				Model 3	0.88 (0.58–1.35)	0.569
MACE	OMT‐nCTO	52/205	25.4	Reference	1.00	—
	ICR‐nCTO	35/155	22.6	Model 1	0.91 (0.59–1.40)	0.679
				Model 2	0.91 (0.59–1.40)	0.677
				Model 3	0.80 (0.51–1.24)	0.320
	CR‐nCTO	37/153	24.2	Model 1	0.94 (0.61–1.43)	0.761
				Model 2	0.93 (0.61–1.42)	0.730
				Model 3	0.75 (0.47–1.19)	0.218

*Note:* Model 1, unadjusted; Model 2, adjusted for age and sex; Model 3, adjusted for clinically relevant baseline variables.

Abbreviations: CI, confidence interval; HR, hazard ratio; CR‐nCTO, complete revascularization of non‐CTO lesions; ICR‐nCTO, incomplete revascularization of non‐CTO lesions; OMT‐nCTO, optimal medical therapy for non‐CTO lesions.

## 4 Discussion

In this multicenter retrospective cohort study of patients with NSTEMI, MVD, and concomitant CTO, complete revascularization involving successful CTO recanalization was associated with lower risks of MACCE and MACE. By contrast, among patients in whom CTO recanalization was not achieved, additional revascularization of non‐CTO lesions was not associated with improved mid‐term outcomes. These findings suggest that residual CTO lesions may partly explain the limited prognostic benefit of incomplete revascularization strategies in this high‐risk population.

### 4.1 Clinical Benefit of CTO Revascularization in NSTEMI With MVD

Previous studies have shown that the presence of CTO is associated with adverse outcomes in patients with NSTEMI and MVD [23, 24]. However, whether CTO revascularization is associated with improved prognosis in this population remains uncertain.

In the present study, CR‐CTO was independently associated with lower risks of MACCE and MACE. One possible explanation is that successful CTO revascularization may reduce residual ischemia and improve myocardial perfusion in selected patients. Conversely, persistent CTO may contribute to residual ischemic burden and impaired myocardial reserve despite treatment of non‐CTO lesions.

Our findings are generally consistent with prior observational studies reporting favorable outcomes associated with successful CTO revascularization [25, 26, 27, 28]. However, differences in study populations, procedural strategies, timing of revascularization, and endpoint definitions should be considered when these results are interpreted. The present study adds to the available evidence by focusing specifically on NSTEMI patients with MVD and CTO in a multicenter real‐world setting.

Nevertheless, the benefits of CTO‐PCI should be interpreted cautiously. CTO interventions remain technically demanding procedures associated with prolonged procedural duration, increased radiation exposure, contrast burden, and potential procedural complications. Therefore, revascularization decisions should be individualized according to anatomical complexity, ischemic burden, myocardial viability, clinical status, procedural feasibility, and operator expertise.

### 4.2 Non‐CTO Revascularization When CTO Recanalization Is Not Achieved

A major finding of this study was that, among patients without successful CTO recanalization, the extent of non‐CTO lesion revascularization was not associated with lower risks of MACCE or MACE. Kaplan–Meier analysis and Cox regression showed similar outcomes among patients treated with optimal medical therapy alone, partial revascularization of non‐CTO lesions, or complete revascularization of non‐CTO lesions.

These findings may have important implications for understanding the prognostic role of residual CTO lesions in NSTEMI patients with MVD. Persistent CTO may represent the dominant determinant of residual ischemic burden and impaired myocardial reserve, potentially limiting the clinical benefit obtained from additional PCI of non‐CTO lesions alone. In this context, the prognostic impact of unrevascularized CTO lesions may outweigh the potential benefit derived from more complete treatment of non‐CTO vessels. Previous studies in STEMI populations have similarly suggested that adverse outcomes associated with MVD may be driven predominantly by concomitant CTO lesions rather than by non‐CTO stenoses alone [29, 30, 31]. Our findings extend these observations to NSTEMI patients and further suggest that residual CTO status may substantially influence the effectiveness of incomplete revascularization strategies.

These results should not be interpreted as evidence against non‐CTO lesion revascularization in general. Non‐CTO PCI may still be appropriate for culprit lesion treatment, symptom relief, or reduction of ischemia in selected vessels. Rather, our findings suggest that when CTO lesions remain unrevascularized, more complete treatment of non‐CTO lesions alone may not necessarily translate into improved mid‐term outcomes. Prospective studies are needed to clarify which NSTEMI patients with CTO may benefit most from different revascularization strategies.

### 4.3 Potential Influence of Revascularization Modality

Differences in revascularization strategies and operator expertise may have influenced treatment selection across centers. In the present study, a proportion of patients in the CR‐CTO group underwent CABG. In descriptive analyses (Supporting Information S1: Table 2), patients treated with CABG had higher rates of adverse events than those treated with PCI. However, these findings should be interpreted with caution, as patients in the CABG group had a higher burden of comorbidities and more complex coronary anatomy, including a greater prevalence of diabetes, chronic kidney disease, three‐vessel disease, and left main involvement. Therefore, the observed differences in outcomes are likely to reflect baseline clinical and anatomical differences rather than the independent effect of the revascularization modality itself. These results should not be interpreted as evidence of superiority of PCI over CABG. Treatment decisions should remain individualized according to anatomical complexity, clinical status, surgical risk, procedural feasibility, and operator or institutional expertise.

### 4.4 Limitations

Several limitations should be acknowledged. First, this was a retrospective observational study, and selection bias and residual confounding could not be fully avoided despite multivariable adjustment. Second, detailed anatomical and procedural data, including lesion complexity, SYNTAX score, ischemic burden, and myocardial viability, were not systematically available. These factors may have influenced both treatment selection and clinical outcomes. Third, patients who died during the index hospitalization were excluded because the study focused on post‐discharge mid‐term outcomes; this may have introduced survivor bias. Fourth, the follow‐up duration was relatively limited, and longer‐term outcomes require further investigation. Finally, because this study was conducted in tertiary centers in China, the findings may not be fully generalizable to other healthcare systems or lower‐volume centers.

## 5 Conclusion

In patients with NSTEMI, MVD, and CTO, complete revascularization involving successful CTO recanalization was associated with lower risks of MACCE and MACE. In contrast, when CTO lesions remained unrevascularized, additional revascularization of non‐CTO lesions alone was not associated with improved mid‐term outcomes. These findings support the need to consider CTO revascularization status when evaluating revascularization strategies in this patient population.

## Author Contributions

Chongyou Rao and Bing Han conceived and designed the study. Wei Liu, Yuling Li, and Chongyou Rao performed the statistical analyses and interpreted the results. Wei Liu drafted the manuscript. Wei Qin, Jixian Gao, and Bo Liu contributed to data collection, data management, and follow‐up. Bing Han and Chongyou Rao critically revised the manuscript for important intellectual content and supervised the study. All authors reviewed and approved the final manuscript and agreed to be accountable for all aspects of the work.

## Ethics Statement

This multicenter retrospective study was conducted in accordance with the principles of the Declaration of Helsinki and was approved by the Biomedical Research Ethics Review Committee of Xuzhou Central Hospital (approval No. XZXY‐LJ‐20200415‐074). The requirement for written informed consent was waived by the ethics committee because of the retrospective observational design of the study and the use of anonymized clinical data.

## Conflicts of Interest

The authors declare no conflicts of interest.

## Supporting information


Supporting File

## Data Availability

The data that support the findings of this study are available from the corresponding author upon reasonable request, subject to institutional and ethical restrictions.
