# Supplementary material for: Mid‐Term Clinical Outcomes According to CTO Revascularization Status in Patients With NSTEMI and Multivessel Disease
Source: Clin Cardiol. 2026 Oct 22;49(10):e70491. doi: 10.1002/clc.70491 (PMC13688248; doi:10.1002/clc.70491)
Supplement: Supplementary file 1 [file CLC-49-e70491-s001.docx]

Supplementary Table S1. Baseline characteristics of subgroups within the ICR-CTO population according to non-CTO revascularization status

| **Variable** | **Overall (n = 513)** | **OMT-nCTO (n = 205)** | **ICR-nCTO (n = 155)** | **CR-nCTO (n = 153)** | **P value** |
| --- | --- | --- | --- | --- | --- |
| **Demographics** |  |  |  |  |  |
| Age, years [median (IQR)] | 67.0 (57.0–74.0) | 68.0 (60.0–74.0) | 67.0 (57.0–74.0) | 65.0 (56.0–73.0) | 0.116 |
| Male, n (%) | 365 (71.2) | 143 (69.8) | 112 (72.3) | 110 (71.9) | 0.849 |
| **Comorbidities** |  |  |  |  |  |
| Hypertension, n (%) | 335 (65.3) | 139 (67.8) | 98 (63.2) | 98 (64.1) | 0.617 |
| Type 2 diabetes, n (%) | 218 (42.5) | 86 (42.0) | 70 (45.2) | 62 (40.5) | 0.698 |
| Dyslipidemia, n (%) | 291 (56.7) | 110 (53.7) | 101 (65.2) | 80 (52.3) | 0.039 |
| Chronic kidney disease, n (%) | 108 (21.1) | 48 (23.4) | 34 (21.9) | 26 (17.0) | 0.320 |
| **Laboratory Parameters** |  |  |  |  |  |
| eGFR, mL/min/1.73 m² [median (IQR)] | 73.5 (57.2–91.8) | 73.3 (55.1–89.0) | 69.0 (53.6–94.1) | 78.1 (64.7–94.0) | 0.090 |
| Total cholesterol, mmol/L [median (IQR)] | 4.04 (3.31–4.80) | 4.16 (3.37–5.05) | 4.16 (3.32–4.96) | 3.87 (3.24–4.48) | 0.029 |
| LDL-C, mmol/L [median (IQR)] | 2.50 (1.91–3.17) | 2.53 (1.94–3.26) | 2.64 (1.92–3.38) | 2.33 (1.90–2.84) | 0.016 |
| **Cardiac Function** |  |  |  |  |  |
| LVEF, % [median (IQR)] | 53.0 (44.0–58.0) | 52.0 (43.0–57.0) | 54.0 (45.0–59.0) | 54.0 (46.0–58.0) | 0.098 |
| GRACE score [median (IQR)] | 146 (126–170) | 146 (126–170) | 147 (125–173) | 142 (122–162) | 0.222 |
| **Angiographic Characteristics** |  |  |  |  |  |
| Two-vessel disease, n (%) | 179 (34.9) | 61 (29.8) | 0 (0.0) | 118 (77.1) | <0.001 |
| Three-vessel disease, n (%) | 334 (65.1) | 144 (70.2) | 155 (100.0) | 35 (22.9) | <0.001 |
| Coronary calcification, n (%) | 102 (19.9) | 57 (27.8) | 26 (16.8) | 19 (12.4) | 0.001 |
| Left main stenosis ≥50%, n (%) | 65 (12.7) | 37 (18.0) | 18 (11.6) | 10 (6.5) | 0.005 |
| **Medications at Discharge** |  |  |  |  |  |
| Clopidogrel, n (%) | 298 (58.1) | 142 (69.3) | 94 (60.6) | 62 (40.5) | <0.001 |
| Ticagrelor, n (%) | 208 (40.5) | 58 (28.3) | 60 (38.7) | 90 (58.8) | <0.001 |
| Nitrates, n (%) | 378 (73.7) | 166 (81.0) | 120 (77.4) | 92 (60.1) | <0.001 |
| β-blockers, n (%) | 394 (76.8) | 156 (76.1) | 131 (84.5) | 107 (69.9) | 0.010 |

**Abbreviations:** CR-nCTO, complete revascularization of non-CTO lesions; eGFR, estimated glomerular filtration rate; ICR-nCTO, incomplete revascularization of non-CTO lesions; IQR, interquartile range; LDL-C, low-density lipoprotein cholesterol; LVEF, left ventricular ejection fraction; OMT-nCTO, optimal medical therapy for non-CTO lesions.

Supplementary Table S2. Baseline characteristics and clinical outcomes according to revascularization strategy

| **Variable** | **PCI (n = 67)** | **CABG (n = 52)** | **P value** |
| --- | --- | --- | --- |
| Follow-up duration, days (mean ± SD) | 522.35 ± 182.77 | 547.17 ± 225.97 | 0.509 |
| Age, years [median (IQR)] | 63.0 (52.0–71.0) | 65.5 (60.8–72.0) | 0.133 |
| Male sex, n (%) | 49 (73.1) | 37 (71.2) | 0.974 |
| Hypertension, n (%) | 35 (52.2) | 37 (71.2) | 0.057 |
| Diabetes mellitus, n (%) | 23 (34.3) | 32 (61.5) | 0.006 |
| Chronic kidney disease, n (%) | 8 (11.9) | 17 (32.7) | 0.011 |
| Left ventricular ejection fraction, % [median (IQR)] | 54.5 (43.8–58.0) | 55.0 (43.5–59.5) | 0.345 |
| GRACE score [median (IQR)] | 139.0 (121.5–159.0) | 142.0 (119.8–161.5) | 0.566 |
| Three-vessel disease, n (%) | 44 (65.7) | 42 (80.8) | 0.106 |
| Left main stenosis ≥50%, n (%) | 2 (3.0) | 9 (17.3) | 0.018 |
| CTO location, n (%) |  |  | 0.815 |
| LAD | 24 (35.8) | 16 (30.8) |  |
| RCA | 24 (35.8) | 19 (36.5) |  |
| LCX | 19 (28.4) | 17 (32.7) |  |
| Diuretics at discharge, n (%) | 19 (28.4) | 25 (48.1) | 0.044 |
| Nitrates at discharge, n (%) | 47 (70.1) | 50 (96.2) | 0.001 |
| ACEI/ARB at discharge, n (%) | 36 (53.7) | 9 (17.3) | <0.001 |
| β-blockers at discharge, n (%) | 57 (85.1) | 37 (71.2) | 0.105 |
| MACCE, n (%) | 2 (3.0) | 12 (23.1) | 0.002 |
| MACE, n (%) | 1 (1.5) | 8 (15.4) | 0.013 |

**Notes:** Comparisons between PCI and CABG should be interpreted descriptively because treatment allocation was non-randomized.

**Abbreviations:** CABG, coronary artery bypass grafting; CTO, chronic total occlusion; MACE, major adverse cardiovascular events; MACCE, major adverse cardiovascular and cerebrovascular events; PCI, percutaneous coronary intervention.
